# *IFNL4* ss469415590 Variant Shows Similar Performance to rs12979860 as Predictor of Response to Treatment against Hepatitis C Virus Genotype 1 or 4 in Caucasians

**DOI:** 10.1371/journal.pone.0095515

**Published:** 2014-04-18

**Authors:** Luis M. Real, Karin Neukam, Rocío Herrero, Josep M. Guardiola, Thomas Reiberger, Antonio Rivero-Juarez, Juliana Salazar, Mattias Mandorfer, Dolores Merino, Vicente Soriano, Antonio Rivero, Juan Macías, Juan A. Pineda, Antonio Caruz

**Affiliations:** 1 Unidad Clínica de Enfermedades Infecciosas y Microbiología. Hospital Universitario de Valme, Sevilla, Spain; 2 Instituto de Biomedicina de Sevilla (IBIS), Sevilla, Spain; 3 Unidad de Inmunogenética, Universidad de Jaén, Jaén, Spain; 4 Servicio de Medicina Interna. Hospital de la Santa Creu i Sant Pau, Barcelona, Spain; 5 Division of Gastroenterology and Hepatology, Department of Internal Medicine III, Medical University of Vienna, Vienna, Austria; 6 Vienna HIV & Liver Study Group, Vienna, Austria; 7 Unidad de Enfermedades Infecciosas, Hospital Universitario Reina Sofía, Córdoba, Spain; 8 Instituto Maimónides de Investigación Biomédica de Córdoba (IMIBC), Córdoba, Spain; 9 Unidad de Enfermedades Infecciosas. Hospital Juan Ramón Jiménez, Huelva, Spain; 10 Departamento de Enfermedades Infecciosas. Hospital Carlos III, Madrid, Spain; CAEBi, Spain

## Abstract

**Objectives:**

The rs12979860 variant, linked to *IL28B* gene, predicts sustained viral response (SVR) to pegylated-interferon/ribavirin (pegIFN/RBV) therapy in Hepatitis C Virus genotype 1 or 4 (HCV-1/4)-infected patients. Recently, a functional variant, ss469415590, in linkage disequilibrium (LD) with rs12979860, has been discovered. Our objective was to assess the value of ss469415590 to predict SVR to pegIFN/RBV in Caucasian HCV-1/4-infected individuals and to compare its performance with that of rs12979860.

**Methods:**

272 Caucasian HCV-1/4-infected patients who completed a course of pegIFN/RBV were genotyped for both rs12979860 and ss469415590 markers. Logistic regression models including factors with univariate association with SVR and each genetic marker were elaborated. The area under the receiver operating-characteristic curve (AUROC) was calculated for each model and both were compared.

**Results:**

Both markers were in LD (r^2^ = 0.82). For rs12979860, 66 (64.0%) CC versus 56 (33.1%) T allele carriers achieved SVR (Adjusted OR = 4.156, 95%CI = 2.388–7.232, p = 4.647×10^−7^). For ss469415590, 66 (66.0%) TT/TT versus 56 (32.5%) –G allele carriers (Adjusted OR = 4.783, 95%CI = 2.714–8.428, p = 6.153×10^−8^) achieved SVR. The AUROC of the model including rs12979860 was 0.742 (95%CI = 0.672–0.813) and of that based on ss469415590 was 0.756 (95%CI = 0.687–0.826) (p = 0.780).

**Conclusions:**

The ss469415590 variant shows an equivalent performance to predict SVR to pegIFN/RBV than the rs2979860 in Caucasian HCV-1/4-infected patients.

## Introduction

Since the association of the rs12979860 polymorphism with viral clearance and response to pegylated interferon and ribavirin (pegIFN/RBV) treatment was reported [1], the genotype of this marker has been widely used as a pharmacogenetic tool. rs12979860 genotyping provides useful information for individualizing the management of patients with chronic hepatitis C [2]. This genetic marker mainly predicts the outcome of therapy of HCV genotype 1 or 4 (HCV-1/4)-infections [3], [4], both in HCV-monoinfected and in HIV/HCV-co-infected individuals [5], [6]. Moreover, this pharmacogenetic marker continues showing impact on the outcome of direct-acting antiviral (DAA)-based regimens, particularly in treatment-naïve patients [7], [8].

The rs12979860 genetic marker is located upstream of *IL28B* gene (also known as *IFNL3* gene) suggesting a prominent role of this locus in the processes involved in viral clearance [1]. IL28B has been implicated in the inhibition of HCV replication in vitro and in vivo [9]–[15]. In addition, it also shows a synergistic anti-HCV effect in combination with IFN-alpha [16]. However, rs12979860 variant has not been consistently associated with hepatic IL28B mRNA expression [17]–[19]. That suggests that this polymorphism should be in linkage disequilibrium (LD) with other functional variants.

Recently, a new variant, denoted ss469415590 (TT/−G) (rs368234815), in strong LD with rs12979860, has been reported, which seems to have functional effects on IL28B expression [20]. Thus, the ss469415590 TT/TT carriers show higher expression of IL28B than –G carriers, independently of the rs12979860 genotype [20]. Interestingly, it has also been found that the –G allele of this new variant introduces a frame shift in the DNA sequence, which induces mRNA expression of an IFN analogue (IFNL4) in stimulated human hepatocytes [21]. In fact, both ss469415590 and rs12979860 polymorphisms are located within the *IFNL4* gene [21]. IFNL4 could limit the action of type I and type III IFNs, reducing the capacity of HCV clearance [21]. According to these findings, ss469415590 might be a better predictor of sustained viral response (SVR) than the rs12979860 variant, but only in African populations, where both markers are not so strongly linked [21]. However, other authors have reported differences between these markers in the performance to predict SVR among Caucasians populations [20].

The contradictory results observed in Caucasians require new validation studies in order to clarify and validate which genetic marker yields the best performance to predict SVR to IFN-based therapies in this population. Considering the relevance of rs12979860 genotype in treatment decisions in HCV-infected patients [2], it is necessary to clarify if this pharmacogenetic tool should be replaced by the ss469415590 genotyping in our geographical area.

The objective of this study was to assess the value of ss469415590 genotyping to predict SVR to pegIFN/RBV among Caucasian HCV-1/4-infected individuals and to compare its performance to that of rs12979860 testing.

## Patients and Methods

### Ethics Statement

This study was in compliance with the national legislation and it was performed according to the ethical guidelines of the Declaration of Helsinki. The study was approved by the Ethics Committee of the Hospital Universitario de Valme. Written consent was obtained from all individuals before sampling.

### Patients and Follow Up

This study was carried out on Caucasian HCV-1/4-infected patients who consecutively completed a course of pegIFN/RBV treatment at the infectious diseases units of six hospitals in Spain and one in Austria between May 2001 and December 2011. Patients were prospectively followed and all of them were seen, at least, at week 4, 12, 24 and 48 while on treatment and 24 weeks after completing therapy. Clinical, biochemical and hematological evaluations were performed at each visit. In all patients, a whole blood sample was collected, frozen and stored for subsequent genetic determinations. Patients in whom SVR could be assessed as an on-treatment setting, i.e. excluding those who voluntarily dropped out or discontinued therapy due to adverse events, were included in this study.

### Treatment Strategies and Response Definition

pegIFN alfa-2a or alfa-2b was administered at doses of 180 µg or 1.5 µg/kg once weekly, respectively, in combination with weight-adjusted RBV (1000 mg/day for <75 kg and 1200 mg/day for ≥75 kg). The scheduled treatment duration was 48 weeks for all patients. SVR was defined as undetectable plasma HCV-RNA 24 weeks after the completion of treatment.

### Genotyping

DNA was extracted from frozen whole blood samples using the Quick Pure Blood DNA extraction Kit (Macherey-Nagel, Düren, Germany) or Magna Pure system (Roche Diagnostics, Mannheim, Germany). The rs12979860 and ss469415590 genetic markers were genotyped by real-time PCR techniques as previously reported [5], [21]. Samples that showed genetic recombination between these markers were re-genotyped for both markers as a quality control.

### Statistical Analyses

The online resource at the Institute for Human Genetics, Munich, Germany (http://ihg.gsf.de) was used for the comparison of rs12979860 and ss469415590 genotypic frequencies between groups to determine p values, odds-ratios (OR) and confidence intervals (C.I.). This resource was also used to carry out the Hardy-Weinberg equilibrium (HWE) test and to calculate the allelic frequencies for each marker. Pair-wise LD estimate (r^2^) was determined using the Plink software [22].

Group comparisons of categorical variables were performed using Pearson chi-square test or Fisher’s exact test. To compare age among groups we used the Mann-Whitney U tests (data not normally distributed). Multivariate logistic regression models were elaborated including variables associated with SVR in univariate analysis (p<0.05), as well as age and gender, to obtain adjusted p and OR values. All these calculations, as well as those performed to obtain the area under the receiver operating-characteristic curve (AUROC) of models, were carried out using the SPSS software 19.0 (IBM Corporation, Somers, NY, USA). Comparisons between the AUROCs were performed using the MedCalc Statistical Software version 12.7.7 (MedCalc Software bvba, Ostend, Belgium; http://www.medcalc.org; 2013).

## Results

### Features of the Studied Population

Two-hundred and seventy-two HCV-1/4-infected individuals, most of them men (79.4%), were included in this study. Among them, 209 (76.8%) patients were co-infected with HIV. The main characteristics of the population studied are depicted in table 1.

**Table 1:** Main characteristics of the study population.

Variables	
**Age** 1 **, years**	43.0 (38.7–46.7)*
**Male gender, no. (%)**	216 (79.4)
**HIV infection, no. (%)**	209 (76.8)
**HCV genotype 4, no (%)**	56 (20.6)
**BMI, Kg/m^2^**	23.7 (21.9–26.5)*
**Plasma HCV RNA, log_10_** **IU/mL**	6.2 (5.6–6.7)*
**Advanced fibrosis** 1 **, no (%)**	97 (39.3)

BMI; body mass index.

*Median (quartile 1–quartile 3).

1 Determined by liver biopsy (F≥3 according to the Scheuer Index) or a liver stiffness value ≥11 kPa.

### General Genotyping Data Analysis

rs12979860 and ss469415590 were in LD (r^2^ = 0.82). Consequently, the minor allelic frequency was similar for both markers (0.38 for rs12979860 T allele and 0.39 for ss469415590–G allele). The rs12979860 genotypes were distributed as follow: 37 (13,6%) TT, 132 (48.5%) CT and 103 (37,9%) CC. The ss469415590 genotypic distribution was 39 (14.3%) –G/−G, 133 (48.9%) TT/−G and 100 (36.7%) TT/TT. Both genotypic distributions were in accordance with the HWE law (p>0.500 in both cases). Fifteen individuals (5.5%) carrying one of the favorable genotypes (rs12979860 CC or ss469415590 TT/TT) showed genetic recombination between these markers.Thus, 6 of them were rs12979860 CT ss469415590 TT/TT and, 9 individuals were rs12979860 CC ss469415590 −G/−G or TT/−G.

### Response to Therapy

One hundred and twenty two patients (44.8%) achieved SVR. The median age (quartile 1- quartile 3) of these patients was 42.0 (37.8–46.6) years versus 43.0 (38.7–46.7) years of those who did not reach SVR (p = 0.263). The rates of patients achieving SVR according to the age, gender, HIV coinfection, viral genotype, body mass index, baseline viral load and advanced fibrosis are depicted in table 2.

**Table 2:** Univariate and multivariate analysis (one model for each genetic marker) of factors associated with SVR.

Variables			Univariate	rs12979860 model	ss469415590 model
		SVR n (%)	p value	AOR (95% CI)/p value	AOR (95% CI)/p value
**Age**	**≤43.0 years**	65 (48.1)	0.214	0.992 (0.956–1.029)/0.677	0.990(0.954–1.027)/0.591
	**>43.0 years**	54 (40.6)			
**Gender**	**Male**	98 (45,4)	0.736	0.855 (0.442–1.654)/0.642	0.792 (0.407–1.543)/0.494
	**Female**	24 (42,9)			
**HIV infection**	**No**	36 (57.1)	0.025	0.461 (0.245–0.866)/0.016	0.443 (0.235–0.838)/0.012
	**Yes**	86 (41,1)			
**HCV genotype**	**1**	94 (43.5)	0.385	NI	NI
	**4**	28 (50.0)			
**BMI**	**<25 Kg/m^2^**	61 (44.9)	0.241	NI	NI
	**≥25 Kg/m^2^**	44 (53)			
**Plasma HCV ARN**	**≤6**×**10^5^ IU/ml**	49 (57.0)	0.004	0.526 (0.371–0.745)/2.923×10^−4^	0.522 (0.367–0.742)/2.903×10^−4^
	**>6**×**10^5^ IU/mL**	67 (38,1))			
**Advanced fibrosis**	**No**	71 (47.3)	0.207	NI	NI
	**Yes**	38 (39.2)			
**rs12979860** †	**TT**	10 (27.0)			
	**CT**	46 (34,8)	*6.459×10^−7^	*4.156 (2.388–7.232)/*4.647×10^−7^	NI
	**CC**	66 (64.0)			
**ss469415590** ‡	**−G/−G**	10 (25.6)			
	**TT/−G**	46 (34.6)	*8.940×10^−8^	NI	*4.783 (2.714–8.428)/*6.153×10^−8^
	**TT/TT**	66 (66.0)			

SVR, Sustained viral response; BMI, body mass index; NI, not included in the model; CI, confidence interval; AOR, adjusted odds ratio.

* p values and AOR are for a recessive model (CC vs CT+TT for rs12979860 and TT/TT vs TT/−G+−G/−G for ss469415590).

† Count for non responders were 27, 86 and 37 for TT, CT and CC genotypes respectively.

‡ Counts for non responders were 29, 87 and 34 for –G/−G, TT/−G and TT/TT genotypes respectively.

**Figure 1:**
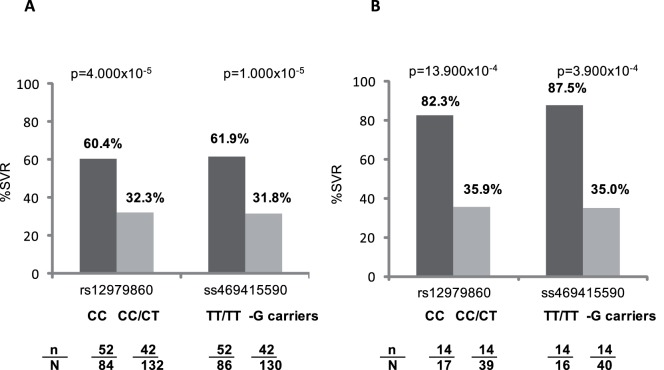
Rates of SVR according to marker genotypes. A: in HCV-1 infected patients and B: in HCV-4 infected patients. n/N: number of patients who achieved SVR/total number of patients (for each genotype group). For each polymorphism, the p values depicted were obtained from the comparison of SVR rates between genotype groups.

For rs12979860, 66 (64.0%) CC versus 56 (33.1%) T allele carriers achieved SVR (OR = 3.599, 95% CI = 2.152–6.021, p = 6.459×10^−7^). For ss469415590, 66 (66.0%) TT/TT versus 56 (32.5%) –G allele carriers showed SVR (OR = 4.021, 95% CI = 2.385–6.780, p = 8.940×10^−8^) (Table 2). Figure 1 shows the rates of SVR according to genetic markers in groups of patients stratified by HCV genotype.

Regarding to the recombinant individuals, 3 (33.3%) among 9 patients bearing the rs12979860 CC genotype, and 3 (50%) among 6 patients bearing the ss469415590 TT/TT genotype achieved SVR (p = 0.622).

### Predictive Performance of Markers

**Figure 2:**
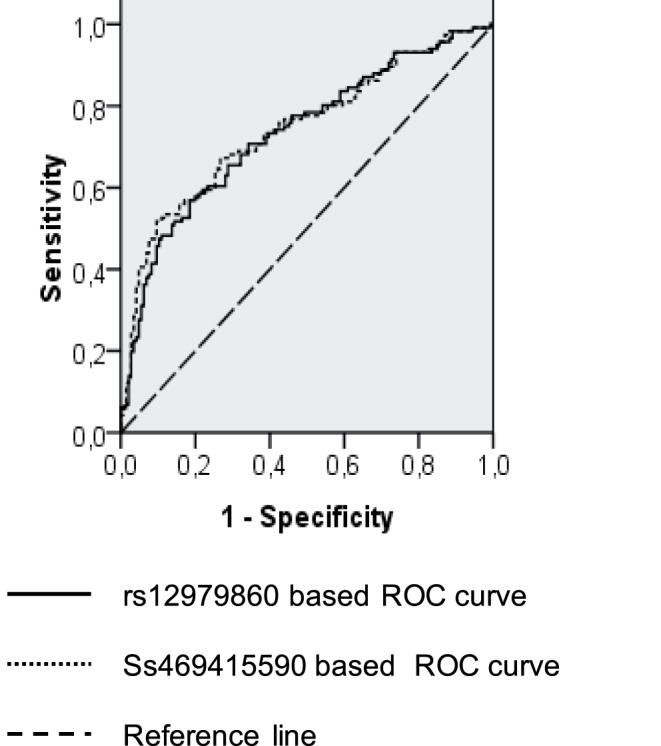
Receiver operating-characteristic curves obtained from each logistic regression model.

The univariate and multivariate associations between the well-stablished predictive factors, plus age and gender, as well as the analyzed genetic factors and SVR are shown in Table 2. The ROCs of the models obtained with each marker are displayed in Figure 2. HIV coinfection, HCV-RNA viral load and the genetic marker included were independently associated with SVR in both models (Table 2). The AUROC of the model that included the rs12979860 marker was 0.742 (95% CI = 0.672–0.813) and of that based on ss469415590 was 0.756 (95% CI = 0.687–0.826) (Figure 2). Both AUROCs were comparable (p = 0.780).

In order to investigate whether these markers could have a different performance as SVR predictors in patients infected with HCV-1 and HCV-4, separate substudies were carried out in these particular subsets. Logistic regression models for each marker, introducing the same variables as in the overall study, were constructed. In the HCV-1 infected patient subgroup the adjusted OR (AOR) (95% CI) obtained for rs12979860 was 3.491 (1.899–6.419) (p = 5.734×10^−5^), and that for ss469415590 was 3.935 (2.118–7.310) (p = 1.460×10^−5^). In the HCV-4 infected patient subgroup, these values were 18.802 (3.342–105.791) (p = 8.725×10^−4^) for rs12979860 and 29.147 (4.215–201.559) (p = 6.305×10^−4^) for ss469415590.

In the HCV-1 infected patient subgroup, The AUROC of the model including the rs12979860 marker was 0.717 (95% CI = 0.656–0.798) and of that including ss469415590 was 0.726 (95% CI = 0.656–0.797). In the HCV-4 infected patient subgroup, these values were 0.830 (95% CI = 0.716–0.945) and 0.847 (95% CI = 0.736–0.958), respectively. There were not significant differences between the AUROCs obtained for each marker neither in the HCV-1 infected patient subgroup (p = 0.859) nor in the HCV-4 infected patient subgroup (p = 0.833).

## Discussion

Our results indicate that ss469415590 and rs12979860 variants perform similarly to predict SVR to pegIFN/RBV in Caucasian HCV-1/4-infected patients. This finding implies that replacing rs12979860 genotype determination with ss469415590 as pharmacogenetic predictive tool in clinical practice would not provide further benefit.

The ss469415590 TT allele seems to increase the IL28B expression [20], and also seems to prevent the IFNL4 expression [21]. Both effects are involved in the inhibition of HCV replication. By the contrary, rs12979860 has not a known functional effect. Accordingly, a higher predictive performance of ss469415590 TT was expected. However, the results of this study do not confirm such a hypothesis.

Both ss469415590 and rs2979860 markers are located within the exon 1 and intron 1 of *IFNL4* gene respectively, separated by 367 pairs of bases and, therefore, in high LD. However, some inter ethnic variations exist in the correlation level between ss469415590 TT and rs2979860 C alleles with stronger correlation in Caucasian than in Africans [21]. This data might explain why we did not observe any differences in the performance of these markers to predict SVR in our Caucasian population, as it has also previously been reported [21], and why these differences are found in Africans [21]. Similar conclusions were obtained by Stattermayer et al. [23] recently. They studied a large sample of HCV monoinfected patients, mainly Caucasians, and concluded that there is no benefit in additional testing for ss469415590 for treatment prediction in these patients. In spite of this fact, a study conducted in the Swiss Hepatitis C Cohort, which included Caucasians HCV-1/4 and HCV-2/3 infected patients [20], reported small, but statistically significant, differences between these markers for predicting SVR achievement. Two additional studies reported contradictory results in Caucasian HIV-HCV-1/4 coinfected patients [24], [25], although none of these studies carried out a formal statistical comparison of the results obtained with each marker. Taken together, all these data suggest that the difference between these markers in terms of performance to predict SVR in Caucasians, if any, is negligible.

Because both markers are in high LD, the possible differences in the performance to predict SVR may be more evident in the recombinant individuals. In accordance with the possible functional role of the ss469415590, a higher rate of SVR was expected in the recombinant individuals bearing the favorable ss469415590 TT/TT genotype than in those bearing the rs12979860 CC genotype. In our study, 15 individuals were recombinants, more than those reported by Staettermayer et al. [23] in their larger sample. In spite of this, that assumption was not proven in the population analyzed herein.

The impact of ss469415590 variations on the likelihood of achieving SVR had been proven in patients bearing HCV-1/4. However, there was little information on subjects infected with HCV-4 so far [23]. This study confirms that ss469415590 genotype has also a robust impact on the response to therapy to pegIFN plus RBV in subjects harboring HCV-4, which might be even stronger than in patients infected with HCV-1 (figure 1). This fact is in agreement with that found with rs12979860 genotype here and in previous studies, where it has been found that this SNP is a reliable SVR predictor in HCV-4 infection [26], with an impact even stronger than on patients infected with HCV-1. But again, ss469415590 genotyping did not show a better performance than rs12979860 variation in HCV-4 carriers either.

This study has two limitations. First, the population analyzed is mostly made up by HIV coinfected patients. Consequently, our results could be mainly applicable to this population. However, as the population analyzed here is the largest HIV/HCV 1/4-coinfected patients reported so far, this study provides the strongest evidence to date on the fact that ss469415590 TT/TT genotype does not perform better than rs12979860 genotype for SVR prediction in this specific subset. Second, this study has been performed on pegIFN/RBV treated patients. This regimen is being replaced with DAA-based combinations. Moreover, the interferon-free regimens will likely be the standard of care in the next few years. Because of this, it could be thought that the future relevance of these findings might be low. However, it has been shown that rs12979860 genotype also impacts on the outcome of most DAA-based regimens including pegIFN [7]. Also, rs12979860 genotype has a role in the viral kinetics and response in interferon-free regimens [8]. These facts suggest that our conclusions could be extended to scenarios of DAA-based therapy.

In conclusion, our results indicate that the ss469415590 marker shows equivalent performance to predict SVR to pegIFN/RBV than the rs12979860 variant in Caucasian HCV-1/4-infected patients. The strong LD between both genetic variants may explain why the possible functional one, i.e. ss469415590, does not perform better than the rs2979860 marker. Consequently, there is no evidence to support a replacement of rs12979860 by ss469415590 genotyping in clinical practice.
